# Host–Microbiota–Parasite Interactions in Grass Carp: Insights from *Ichthyophthirius multifiliis* Infection

**DOI:** 10.3390/microorganisms13040872

**Published:** 2025-04-10

**Authors:** Fangxiang Li, Dongdong Jiang, Qing Wang, Ouqin Chang, Jiyuan Yin, Meiling Yu, Houjun Pan

**Affiliations:** 1 College of Animal Science and Technology, Guangxi University, Nanning 530004, China; 2 Key Laboratory of Fishery Drug Development, Ministry of Agriculture and Rural Affairs, Guangdong Provincial Key Laboratory of Aquatic Animal Immunology and Sustainable Aquaculture, Pearl River Fisheries Research Institute, Chinese Academy of Fishery Sciences, Guangzhou 510380, China

**Keywords:** ciliate parasite, microbiome dysbiosis, mucosal immunity, opportunistic bacteria, parasitosis, fish

## Abstract

The ciliate parasite *Ichthyophthirius multifiliis* poses significant threats to grass carp (*Ctenopharyngodon idellus*) aquaculture. However, the limited understanding of host microbiota shifts and immune responses hinders effective control strategies. This study integrated analyses of host pathological indices, immune response and skin/gill/gut microbiota shifts after *I. multifiliis* infection. A histopathological examination identified gill and fin tissues embedded with *I. multifiliis*, accompanied by epithelial necrosis, and inflammatory cell infiltration. Biochemical profiling revealed marked elevations in aspartate aminotransferase (AST), alanine aminotransferase (ALT), urea (UREA), and creatinine (CREA) levels, indicating impaired hepatic and renal function. Quantitative RT-PCR analyses demonstrated the up-regulation of mucosal immune gene *IgT* and pro-inflammatory cytokine *TNF-α* while increasing the trend of systemic immune gene *IgM.* 16S rRNA sequencing revealed significant reductions in skin microbiota diversity. At the genus level, opportunistic pathogens *Aeromonas* and *Vibrio* proliferated in the intestine, whereas *Flavobacterium* and *Candidatus* Megaira increased in the skin and gills. Correlation analyses identified positive associations between *Aeromonas/Vibrio* abundance and host phenotype, contrasting with negative correlations observed for *Sphingomonas*, *Acinetobacter*, and *Leifsonia*. These findings demonstrate that *I. multifiliis* infection induces host microbiome dysbiosis and potentially opportunistic bacterial infections. This investigation advances our understanding of tripartite host–microbiota–parasite interactions and supports microbial community-based parasitosis control in fish culture.

## 1. Introduction

Parasitic infections pose a significant threat to intensive aquaculture, impacting both fish health and economic productivity. Traditionally, the control of fish parasitic diseases has heavily relied on chemical treatments, which, while effective, raise concerns over food safety and environmental sustainability [1]. This has spurred a growing interest in developing eco-friendly alternatives for parasite control. Based on the discovery of the “host–microbiota–parasite” interaction mechanism [2,3,4], researchers have initiated the study of an alternative strategy utilizing gut microbes to combat parasitic infections in sheep [5].

While the tripartite interactions among the “host, microbiota, and parasite” have been extensively studied in mammals [2,3,6,7,8], research in fish is still catching up. Nonetheless, there has been a notable increase in studies exploring parasite–microbiota interactions in fish, including those involving the skin microbiota and ectoparasites [4], gut microbiota and intestinal parasites [9,10,11,12], and even gut microbiota and kidney parasites [13]. Additionally, there is evidence that ectoparasites on fish gills can influence the gut microbiota at a distance [14]. Despite these advances, the mechanisms underlying “host–microbiota–ectoparasite” interactions in fish remain poorly understood.

Grass carp (*Ctenopharyngodon idellus*), a cornerstone of freshwater aquaculture in China, plays a dual role in food production and water quality management, making it a globally significant species [15,16]. However, its husbandry is frequently challenged by parasitic infections, particularly by the ectoparasite ciliate *Ichthyophthirius multifiliis*, which inflicts substantial economic losses [17,18,19]. Besides grass carp, *I. multifiliis* infects a wide range of freshwater fish species, including rainbow trout (*Oncorhynchus mykiss*), channel catfish (*Ictalurus punctatus*), and goldfish (*Carassius auratus*) [4,18,19,20,21]. This ectoparasite undergoes a complex life cycle, involving three main stages: invasive theronts in water, feeding trophonts that primarily infest fish gills and skin, and reproductive tomonts that release new infectious larvae. *I. multifiliis*, characterized by its low host specificity and confinement to the surface epithelial cell layers in fish, serves as an ideal model for studying host mucosal immunity and parasite–microbiota interactions [4,20,21]. In rainbow trout, *I. multifiliis* infection triggers mucosal immune responses, including IgT binding and TNF-α up-regulation, alongside microbiota alterations [4,22,23]. Similarly, grass carp exhibit immune activation through Toll-like receptor pathways and cytokine interactions [24,25,26], However, the specific mucosal immune responses and associated microbiota alterations during *I. multifiliis* infection remain poorly understood. Furthermore, the complex mechanisms underlying the tripartite interactions among the host, microbiota, and parasite have yet to be elucidated.

Here, we integrated 16S rRNA sequencing, histopathology, biochemical and qRT-PCR techniques to investigate how the ideal model ectoparasite *I. multifiliis* infection alters the short-range (skin and gill) and long-range (intestine) microbiota in grass carp while assessing associated tissue damage and immune markers. Our findings attempt to enhance the understanding of “host–microbiota–parasite” interactions, providing a possible foundation for microbial-based disease management in aquaculture.

## 2. Materials and Methods

### 2.1. Infection Experiment

All experimental fish protocols were approved by the Laboratory Animal Ethic Committee Pearl River Fisheries Research Institute, CAFS (No.: LAEC-PRFRI-2024-03-44).

#### 2.1.1. Infection of Parasites

##### Parasite-Free Grass Carp

Juvenile grass carp with a body weight of 30~50 g were obtained from the Pearl River Fisheries Research Institute of CAFS in Guangzhou, China. These fish were cultured in several 1000 L aquaria equipped with aerators, maintaining water temperatures at 24.0 ± 2 °C, dissolved oxygen ≥ 5.0 mg/L, ammonia nitrogen concentrations < 0.35 mg/L, and nitrite nitrogen concentrations < 0.01 mg/L. Prior to their introduction into the tanks, the fish underwent three consecutive formalin baths, each involving immersion in a final concentration of 100 × 10^−6^ formalin solution for 1 h, with 48 h intervals between treatments, to eliminate all ecto-parasites. Subsequently, grass carp were randomly selected for testing parasitic, bacterial, and viral infections. For parasite detection, 30 fish were randomly chosen and anesthetized by immersion in 100 mg/L Tricaine methanesulfonate (MS-222, Sigma-Aldrich, St. Louis, MI, USA). Following anesthetization, each sample was thoroughly examined for parasites using a stereomicroscope (Zeiss Axio, Germany, 16× continuous zoom) and a microscope (Nikon Eclipse 80i, Japan) at magnifications of ×40, ×100, ×400, and ×1000. It was confirmed that each fish was free of parasitic infection. For bacterial and viral detection, 10 fish were randomly selected and, after MS-222 anesthesia, were handled on a strictly sterile clean bench. Grass carp reovirus (GCRV) was detected from liver, kidney, and spleen tissues using reverse transcription quantitative real-time PCR (qRT-PCR) methods. For bacterial detection, *Flavobacterium* species from fish gills were selected isolated by using Shieh selective medium [27]. Additionally, 5% blood agar media and nutrient agar were used to isolate and identify bacteria such as *Aeromonas* and *Vibrio* species from liver, spleen, and kidney tissues, according to the methods in the literature [28].

##### Parasitic Infection in Grass Carp

The methods for parasite isolation and infection were based on procedures previously described the literature [26,29]. The serial passage of *I. multifiliis* at the Pearl River Fisheries Research Institute of CAFS were overdosed with the anesthetic MS-222 and placed in a water-filled container to facilitate the excretion of trophonts in the water. After 4 h, the fish were removed from the water and the trophonts remained and were kept at 15 °C ± 1.0 °C for approximately 24 h to form tomonts, which subsequently released theronts.

For parasite infection, grass carp were exposed to an optimal parasitic dose after parasite infective dose pre-screening assays. Preliminary studies of dose–response trials showed that the LD_50_ in grass carp at 14 days of the experiment was approximately 6000 theronts per fish in 600 L water, which was approximately 300 theronts/L water.

##### Experimental Fish Grouping

After the acclimatization/microbial check phases for 2 weeks, 360 grass carp were divided into two groups, 180 in the control (un-infected) group and 180 in the experimental (infected) group, with each group having 6 replicas. The formal assay used 12 tanks (30 fish/tank), and each tank had an aerator and was filled with 600 L filtered water. During the experiment, 30% of the water was changed every day. The fish were fed with a pellet feed (protein content of 28%) every day, and the daily dosage was approximately 1% of their body weight.

In the formal infection experiment, for the infected group, we added approximately 3000 theronts per fish into each tank (150 theronts/L). The control fish (the uninfected group) were fed and maintained similarly but were not exposed to parasites.

For microbiota, serum biochemical, and immune gene expression analyses, we collected 3 fish tissues and mixed them to form 1 sample. For microbiota analyses, for each group, 6 samples were obtained and sequenced on day 11, while for serum biochemical indicators, 8 samples were tested on day 11. This means we used 24 fish in the uninfected and infected group for the serum biochemical indicator test and 18 fish in each group for microbiota analyses. The fish for the immune gene testing were sampled according to the experimental timeline (at days 0, 1, 3, 5, 7, and 11), with each group tested at 6 time points with 6 samples (18 fish) per time point, which means there were 108 fish in each group for the immune gene expression testing.

### 2.2. Sample Collection

For the analyses of biochemical indicators, blood was collected from the caudal vein of each fish using sterile, heparin-free syringes and kept at 4 °C for approximately 12 h to allow serum precipitation. Subsequently, the serum was separated by centrifugation at 3000× *g* for 10 min at 4 °C, and we collected 3 fish sera in one sterile tube, which was stored at −80 °C for further analysis. To analyze microbiota, gill samples, skin mucus samples, and posterior intestinal samples were collected under sterile conditions. Specifically, approximately 0.5 g of gill tissue was collected by using sterile scissors. Skin mucus was collected by scraping the lateral sides of the fish with a sterile scraper. And samples approximately 2.0 cm in length containing intestinal contents were collected by using sterile scissors. Under sterile conditions, each tissue sample from 3 fish was individually placed into sterilized and pre-labeled Eppendorf tubes and stored at −80 °C. For the analyses of immune-related gene expression levels, samples from the gill, skin, and kidney were collected on days 0, 1, 3, 5, 7, and 11 after infection, frozen in liquid nitrogen and stored at −80 °C. Additionally, for histopathological analyses, tissue samples of the gills and skin were collected and fixed in 4% paraformaldehyde.

### 2.3. Biochemical Analysis

To investigate the impact of parasitic infection on the internal organs of grass carp, we measured eight related serum biochemical indicators. These included liver functional parameters—alanine aminotransferase (ALT), aspartate aminotransferase (AST), total bilirubin (TBIL), and lactate dehydrogenase 1 (LDH1); heart functional parameters—creatine kinase (CK), creatine kinase MB isoenzyme (CK-MB), and LDH1; and kidney function parameters—creatinine (CREA) and urea (UREA). All indicators were measured using commercial assay kits (Nanjing Jiancheng Bioengineering Institute, China), following the manufacturer’s instructions.

### 2.4. Histopathological Analysis

Fish tissue samples were fixed in 4% (*w*/*v*) paraformaldehyde for 24 h. Following fixation, the samples underwent dehydration using an ethanol gradient, clearing them with xylene, and were then embedded in paraffin according to standard histological techniques. Sections were cut to a thickness of 5 µm using a rotary microtome (RM2235, Leica Camera Company, Wetzlar, Germany) and mounted onto glass slides. The sections were subsequently stained with hematoxylin and eosin (H&E). Histological samples were observed and photographed using a compound microscope (Olympus BX41, Olympus Corporation, Tokyo, Japan).

### 2.5. Microbiota Analysis

#### 2.5.1. DNA Extraction

Genomic DNA from posterior intestinal, gill, and skin mucus samples was extracted using the Guide S96 Magnetic Soil/Fecal DNA Extraction Kit (Tiangen Biotech Co., Ltd., Beijing, China). DNA concentration was determined using the Qubit™ dsDNA HS Assay Kit and the Qubit 4.0 Fluorometer (Invitrogen, Thermo Fisher Scientific, Waltham, MA, USA).

#### 2.5.2. PCR Amplification

The V3-V4 region of the 16S rRNA gene was amplified from genomic DNA extracted from gill, skin, and posterior intestinal tissues using the universal primers 341F(5′-CCTACGGGNGGCWGCAG-3′) and 805R(5′-GACTACHVGGGTATCTAATCC-3′). To facilitate high-throughput sequencing, sample-specific Illumina index sequences were added to the 5′ ends of both forward and reverse primers. PCR amplification was carried out in a 25 µL reaction mixture containing 50 ng template DNA, 2.5 µL each of forward and reverse primers (10 µM), 12.5 µL Phusion Hot Start Flex 2X Master Mix, and nuclease-free water to reach the final reaction volume. PCR conditions included initial denaturation at 98 °C for 30 s, followed by 35 cycles consisting of denaturation at 98 °C for 30 s, annealing at 54 °C for 30 s, and extension at 72 °C for 45 s, with a final extension step at 72 °C for 10 min. PCR products were visualized on 2% agarose gels to verify successful amplification. Throughout DNA extraction and amplification processes, ultrapure water was used as a negative control instead of DNA samples to rule out false positive results. PCR products were subsequently purified using AMPure XT beads (Beckman Coulter Genomics, Danvers, MA, USA) and quantified using a Qubit fluorometer (Invitrogen, Waltham, MA, USA). The size and concentration of the amplicon libraries were assessed using an Agilent 2100 bioanalyzer (Agilent, Santa Clara, CA, USA) and Illumina library quantification kits (Kapa Biosystems, Boston, MA, USA), respectively. Finally, The libraries were sequenced on the NovaSeq PE250 platform.

#### 2.5.3. Bioinformatics Analysis

Samples were sequenced using the Illumina NovaSeq platform according to the manufacturer’s protocols. Paired-end sequences were sorted based on unique sample barcodes, and the introduced barcode and primer sequences were subsequently trimmed. Overlapping paired-end reads were merged using FLASH software. Raw sequencing reads underwent quality control using fqtrim (version 0.94) under defined filtering parameters to obtain high-quality clean sequences. Chimeric sequences were identified and removed with Vsearch (version 2.3.4). The DADA2 algorithm was employed for sequence denoising, resulting in the generation of a representative feature sequence set and feature abundance tables. Diversity metrics were calculated by normalizing to the same random sequence depth. Taxonomic classifications and relative abundances of microbial features were determined using the SILVA database (release 132). Alpha diversity, representing microbial diversity within samples, was evaluated using the Chao1, Observed Species, Good’s Coverage, Shannon, and Simpson indices through QIIME2 software. Beta diversity was analyzed using QIIME2 and visualized through R packages. Sequence alignment was performed using QIIME2 and lastal+(1.04)BLAST, and each representative sequence was annotated against the SILVA and NT database. Spearman’s rank correlation analyses were conducted to examine associations between microbial taxa and phenotypic traits. Additional figures were produced using R software (version 3.5.2).

### 2.6. Expression Analysis of Selected Immune-Related Genes

Tissues were homogenized and total RNA was isolated using TRIzol LS reagent (Invitrogen, Thermo Fisher Scientific, Waltham, MA, USA) according to the manufacturer’s instructions. Total RNA was incubated with RNase-free DNase I (Roche, Indianapolis, IN, USA) to remove contaminating genomic DNA, and then the RNA samples were reverse transcribed into cDNA using random hexamer primers and Moloney murine leukemia virus (MMLV) reverse transcriptase (Takara, Shiga, Japan) and stored at −20 °C until further analysis.

The immune-related genes determined in our study included those encoded for immunoglobulin T (IgT), immunoglobulin M (IgM),and Tumor Necrosis Factor alpha (TNF-α). The qRT-PCR mixture consisted of 10 μL of 2× SYBR Green PCR master mix (TaKaRa), 7.2 μL of nuclease-free water, 0.4 μL of each gene specific primer (10 mM), and 2 μL of cDNA. The expression levels of immune-related genes were calculated using 2^–ΔΔCT^ to indicate an n-fold difference relative to the calibrator. *β-actin* was used as the reference gene. The primers are listed in Table 1.

### 2.7. Statistical Analysis

Data are presented as mean ± standard deviation (SD). A one-way analysis of variance (ANOVA) was conducted on the three groups of data. If significant differences were detected (*p* < 0.05), Duncan’s multiple range test was employed to rank the means. A *p*-value of <0.05 was considered statistically significant.

## 3. Results

### 3.1. Pathogen Detection Demonstrated That I. multifiliis Successfully Infected Grass Carp, Followed by Secondary Bacteria Invasion

Detailed parasite detection results indicated that no parasites were found in the gills, skin, fins, muscle, eyes, blood, intestine, liver, spleen, and swim bladder of fish in the control (uninfected) group. In contrast, the infected group exhibited only the parasite *I. multifiliis* in the gills, skin, fins, and head eye orbit. Additionally, the detection of grass carp reovirus (GCRV) yielded negative results both in the uninfected and infected groups. Interestingly, the bacteria *(Flavobacterium* and *Aeromonas*) were determined to be positive in the infected group while negative in the control group. The comprehensive pathogen detection results demonstrated that parasite *I. multifiliis* infection was identified, followed by opportunistic pathogens being isolated, thereby confirming that the disease in grass carp was primarily caused by *I. multifiliis* (Table 2).

### 3.2. Histopathology Suggested That the Trophonts of I. multifiliis Could Be Embedded in Epithelial Cells of Infected Fish Gills and Skin

**Figure 1:**
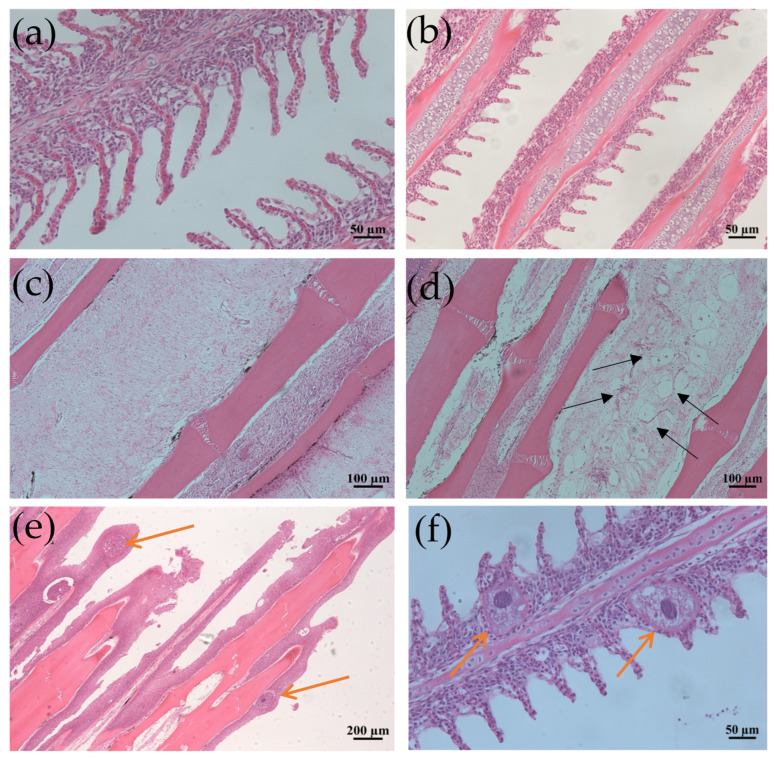
Histological section changes in gills and fins caused by *I. multifiliis* infection in grass carp. (**a**) Normal gill filaments. (**b**) Gill filaments after infection. (**c**) Normal fin rays. (**d**) Fin rays after infection. Black arrows point to the mucous cells. (**e**) Significant epithelial hyperplasia, partial loss of fins due to severe infection. (**f**) Enlarged view of gill filaments infected by *I. multifiliis.* Yellow arrows show trophonts in (**e**,**f**).

Grass carp infected with *I. multifiliis* exhibited notable histopathological alterations compared to the control fish. In histological sections of the fins and gills of the infected fish, parasitic trophonts were observed, typically embedded in epithelial cells. The structural features of the parasites, including large horseshoe-shaped nuclei, cytoplasmic food vacuoles, and superficial cilia, were frequently visible. Parasitism by *I. multifiliis* on the gills led to lamellar deformation, capillary congestion, exudation, or localized ischemia, as well as the swelling and necrosis of respiratory epithelial cells. Additionally, there was the hyperplasia of mucous cells with increased secretion, accompanied by the substantial infiltration of eosinophils and lymphocytes. In cases of severe infection, hyperplasia and inflammatory cell infiltration, along with epithelial cell necro and vacuolar degeneration, were observed (Figure 1).

### 3.3. Serum Biochemical Changes Indicated That I. multifiliis Infection Significantly Induced Hepatic and Renal Injury in Grass Carp

The results of the biochemical indicator examination for grass carp in the *I. multifiliis*-infected group and the uninfected group are presented in Table 3. The findings revealed that grass carp in the infected group exhibited significant differences in multiple physiological indicators. Specifically, the activities of AST and ALT were markedly elevated in the infected group, increasing by 3.85-fold and 2.87-fold, respectively, compared to the uninfected group (*p* < 0.05). This suggests that the infection may have caused liver cell damage. Additionally, the levels of UREA and CREA were significantly higher in the infected group (*p* < 0.05), indicating a potential impact of the infection on the renal function of grass carp. CK and its isoenzyme CK-MB showed a tendency to increase in the infected group but no significant differences with the control group (*p* > 0.05), which might reflect certain influences on heart function after *I. multifiliis* infection. These data indicate that external parasitic infection significantly affects multiple physiological functions in grass carp, particularly with pronounced effects on liver and kidney indicators.

### 3.4. Alterations of the Microbiota in Skin, Gills, and Intestine Indicated I. multifiliis Infection Increased the Abundance of Opportunistic Pathogen Bacteria

16S rRNA sequencing showed that after trimming and filtering, a total of 739,796 high-quality clean reads were obtained from gill samples, 738,993 from intestine samples, and 795,376 from skin samples (with six replicates for each sample). Following the removal of sequences classified as “unknown”, “cyanobacteria”, “chloroplast”, and “mitochondria”, similarity clustering resulted in 1931 Amplicon Sequence Variants (ASVs) in gill samples, 1074 ASVs in intestine samples, and 2369 ASVs in skin samples.

#### 3.4.1. NMDS Analysis

**Figure 2:**
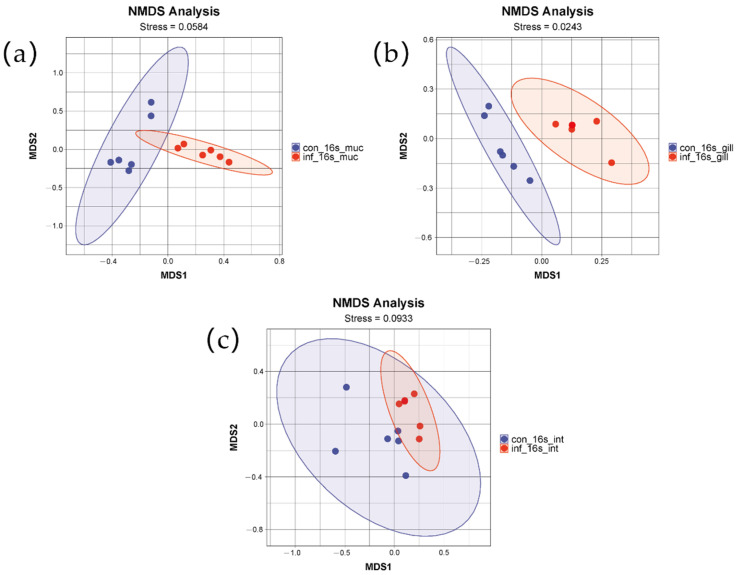
NMDS analysis of skin, gill, and intestinal microbiomes in grass carp after infection with *I. multifiliis.* (**a**) Skin. (**b**) Gill. (**c**) Intestine. Red color indicates samples infected with *I. multifiliis.* Blue color indicates uninfected samples.

To assess the differences in microbial community structure between the control group and the infected group, non-metric multidimensional scaling (NMDS) based on weighted UniFrac distances was employed. The NMDS results for the gills, intestine, and skin, as depicted in the figures, clearly demonstrate significant differences between the two groups (Figure 2). The distributions of the two groups in the two-dimensional space formed two distinctly separated clusters, indicating that the infection had a significant impact on the microbial community structure. The stress values were 0.02, 0.09, and 0.0584, respectively, indicating that the NMDS analyses for the three tissues exhibited good fitting and were capable of accurately reflecting the multidimensional data characteristics of the community structures.

#### 3.4.2. Alpha Diversity of Microbiota

##### Skin

In the skin microbial communities, infection significantly impacted species richness and diversity. The Observed Species decreased from 452.00 ± 10.12 in the control group to 280.17 ± 12.45 in the infected group, the Shannon index from 5.38 ± 0.33 to 3.64 ± 0.45, and the Chao1 index from 455.19 ± 15.45 to 281.92 ± 13.65, all exhibiting significant differences (*p* < 0.05). These results indicated that infection markedly reduced both the number of species and the diversity in the skin microbiota. However, the Simpson index decreased from 0.86 ± 0.05 to 0.73 ± 0.04, which did not reach statistical significance but suggested a reduction tendency in species evenness (Table 4).

##### Gill

The gill microbial communities experienced less pronounced changes compared to the skin and intestines. The Observed Species decreased from 341.00 ± 10.12 in the control group to 321.50 ± 12.45 in the infection group, the Shannon index from 3.93 ± 0.33 to 3.37 ± 0.45, and the Simpson index from 0.70 ± 0.05 to 0.68 ± 0.04. However, these changes did not achieve statistical significance (*p* > 0.05), indicating that infection had a minimal impact on the gill microbial communities (Table 4).

##### Intestine

In the intestinal microbial communities, notably, the Simpson index significantly declined in the infected group (*p* < 0.05), from 0.65 ± 0.04 to 0.52 ± 0.05, indicating that infection substantially reduced the evenness of the intestinal microbiota. the impact of infection on species richness was minimal, with Observed Species slightly decreasing from 177.33 ± 12.45 in the control group to 172.50 ± 10.12, a change that was not statistically significant (*p* > 0.05). The Shannon index decreased from 2.88 ± 0.45 to 2.57 ± 0.33, also without significant difference. The Chao1 index exhibited minimal change, suggesting that infection had a limited effect on the potential species richness in the intestine (Table 4).

#### 3.4.3. Skin Microbiota Diversity Reduced from ASV Distribution Venn Diagram Analysis

**Figure 3:**
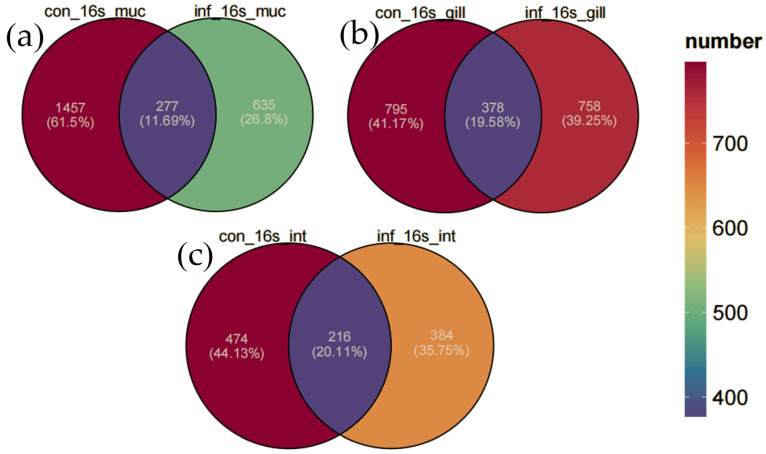
Venn diagram of ASV distribution. (**a**) Skin. (**b**) Gill. (**c**) Intestine.

In this study, Venn diagrams were employed to analyze the changes in the microbial communities of the skin, gills, and intestine of grass carp before and after infection (Figure 3). The microbial communities in the skin of the control group possessed 1457 unique ASVs (61.5%), and the infected group had 835 unique ASVs (35.2%). The results revealed that infection significantly reduced the diversity of the skin microbiota, with a substantial decrease in the number of unique ASVs. However, in contrast, the microbial communities in the gills and intestine remained relatively stable. Specifically, in the gills, the control group contained 795 unique ASVs (41.7%), the infected group had 758 unique ASVs (39.8%), and they shared 378 ASVs (19.5%). In the intestine, the control group featured 474 unique ASVs (44.1%), the infected group had 384 unique ASVs (35.7%), and they shared 216 ASVs (20.1%). Overall, the number of ASVs declined in all three tissues in the infected group, with the sharpest decrease in the skin, a moderate reduction in the intestine, and the least in the gill.

These results indicate that the microbial communities of different tissues in grass carp respond differently to *I. multifiliis* infection. The skin microbiota exhibited the most pronounced changes, while the gill microbiota maintained the highest level of stability despite the infection.

#### 3.4.4. Microbiota Composition Influenced by *I. multifiliis* Infection

##### At the Phylum Level

**Figure 4:**
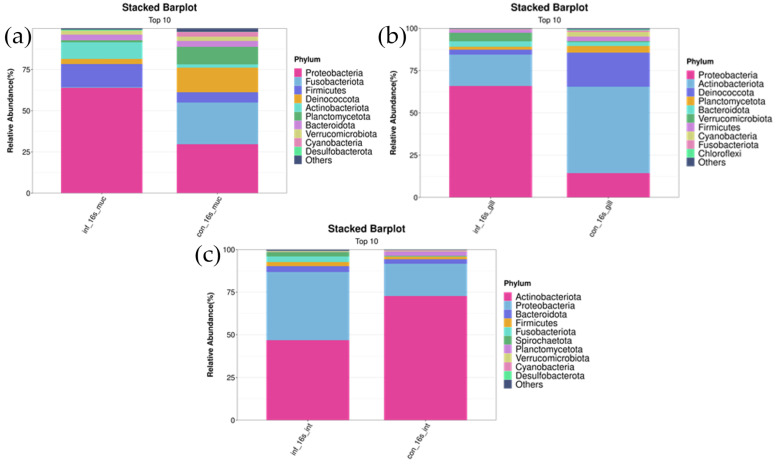
Stacked bar chart of top 10 bacterial phyla in skin, gill, and intestinal microbiomes of *I. multifiliis* infection and control grass carp. (**a**) Skin. (**b**) Gill. (**c**) Intestine.

In the skin, *Proteobacteria*, *Deinococcota*, and *Firmicutes* were the most abundant bacterial phyla in both groups. Compared to the control group, the infected group had a lower abundance of *Deinococcota* (infected group: 3.1 ± 1.9% vs. control group: 14.8 ± 12.0%, *p* < 0.05), while the abundance of *Proteobacteria* was higher (infected group: 64.0 ± 18.2% vs. control group: 29.7 ± 19.0%, *p* < 0.01). As for *Firmicutes*, although the abundance was higher in the infected group compared to the uninfected group (infected group: 14.0 ± 9.5% vs. control group: 6.2 ± 2.5%), the difference did not reach statistical significance (*p* > 0.05) (Figure 4a).

In the gills, *Actinobacteriota* and *Proteobacteria* were the two most abundant bacterial phyla in both the control and infected groups. Compared to the uninfected group, the abundance of *Actinobacteriota* significantly decreased in the infected group (infected group: 18.5 ± 11.3% vs. control group: 51.1 ± 24.1%, *p* < 0.05), whereas the abundance of *Proteobacteria* significantly increased (infected group: 66.0 ± 10.7% vs. control group: 14.5 ± 4.5%, *p* < 0.01) (Figure 4b).

In the intestine, *Actinobacteriota*, *Proteobacteria*, and *Bacteroidota* were the most abundant bacterial phyla in both groups. Compared to the uninfected group, the infected group had a lower abundance of *Actinobacteriota* (infected group: 46.9 ± 31.1% vs. control group: 72.6 ± 9.0%, *p* < 0.01) and a higher abundance of *Proteobacteria* (infected group: 40.0 ± 20.4% vs. control group: 18.9 ± 5.4%, *p* < 0.05). The abundance of *Bacteroidota* did not differ significantly between the infected and uninfected groups (infected group: 2.6 ± 1.9% vs. control group: 3.5 ± 4.1%, *p* > 0.05) (Figure 4c).

##### At the Genus Level

**Figure 5:**
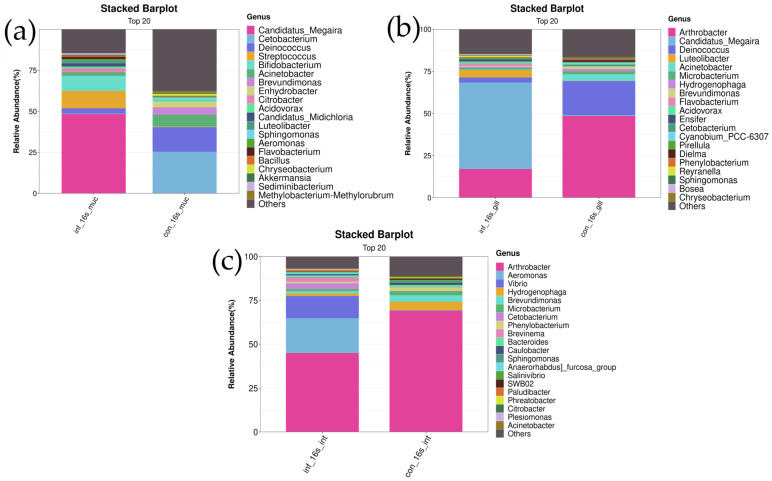
Stacked bar chart of top 20 bacterial genera in skin, gill, and intestine microbiomes of *I. multifiliis* infection and control grass carp. (**a**) Skin. (**b**) Gill. (**c**) Intestine.

In the skin, the genus *Candidatus Megaira* was significantly more abundant in the infected group compared to the control group (infected group: 48.1 ± 20.5% vs. control group: 0.3 ± 0.6%, *p* < 0.01). Additionally, the abundance of the genus *Candidatus Midichloria* was significantly higher in the infected group compared to the control group (infected group: 1.7 ± 1.2% vs. control group: 0.02 ± 0.05%, *p* < 0.05). The abundance of the genus *Flavobacterium* was also significantly higher in the infected group (infected group: 1.7 ± 0.7% vs. control group: 0.01 ± 0.02%, *p* < 0.01) (Figure 5a).

In the gills, the genus *Candidatus Megaira* was significantly more abundant in the infected group compared to the control group (infected group: 50.7 ± 23.6% vs. control group: 0.7 ± 0.5%, *p* < 0.01). The abundance of the genus *Luteolibacter* was also markedly higher in the infected group (infected group: 4.1 ± 3.9% vs. control group: 0.1 ± 0.1%, *p* < 0.05). The abundance of the genus *Flavobacterium* showed a significant difference between the two groups, with the infected group having a higher abundance (infected group: 1.5 ± 2.0% vs. control group: 0.04 ± 0.08%, *p* < 0.01) (Figure 5b).

In the intestines, differences in the relative abundance of the genera *Vibrio* and *Aeromonas* were observed between the control and infected groups. The results showed that the abundance of *Vibrio* was significantly higher in the infected group compared to the control group (infected group: 18.8 ± 19.7% vs. control group: 0.09 ± 0.1%, *p* < 0.05). Similarly, the abundance of *Aeromonas* was also significantly increased in the infected group (infected group: 19.6 ± 24.2% vs. control group: 0.31 ± 0.5%, *p* < 0.05). These results indicate a significant increase in the abundance of *Vibrio* and *Aeromonas* under infection conditions. Additionally, the genus *Brevundimonas* exhibited a significantly lower relative abundance in the infected group compared to the control group (infected group: 1.4 ± 1.0% vs. control group: 3.7 ± 2.1%, *p* < 0.05). Similarly, the abundance of *Hydrogenophaga* was significantly decreased in the infected group (infected group: 1.4 ± 0.9% vs. control group: 4.2 ± 2.3%, *p* < 0.05). These findings suggest that the abundances of these two genera are significantly reduced under infection conditions (Figure 5c).

### 3.5. Functional Prediction of Skin, Gill, and Intestinal Microbiota After I. multifiliis Infection of Grass Carp

**Figure 6:**
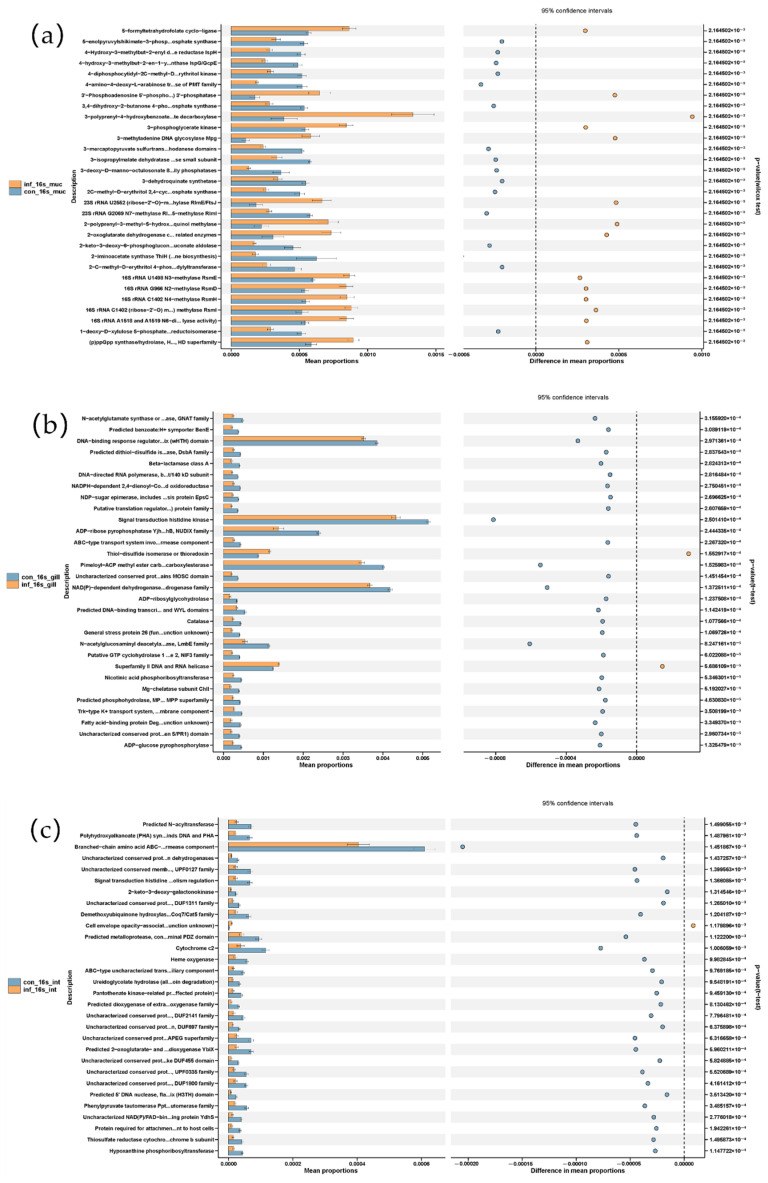
Microbial metabolic shifts in grass carp after *I. multifiliis* infection using PICRUSt prediction. (**a**) Skin. (**b**) Gill. (**c**) Intestine.

PICRUSt-based functional prediction revealed distinct changes in microbial functions across the skin, gill, and intestine of grass carp following *I. multifiliis* infection. In the skin, the infected group showed increased functions of the “bacterial secretion system” and pathways related to “cell division” and “cell cycle—Caulobacter”, while metabolic pathways such as “fatty acid biosynthesis” and “alanine, aspartate and glutama...amate metabolism” were reduced. In the gill, most metabolic pathways showed reduced functions related to, for example, “signal transduction histidine kinase”, “Pimeloyl-ACP methyl ester carb…carboxylesterase”, and “NAD(P)-dependent dehydrogenase…drogenase family”, while elevated levels of “thiol-disulfide isomerase” and “Superfamily II DNA and RNA helicase” were observed in the infected group. In the intestine, most metabolic pathways showed reduced functions, in which the “branched-chain amino acid ABC transporter, permease component” was most prominent and showed a pronounced decrease in the infected group (Figure 6).

Altogether, the functional prediction of the microbiota using PICRUSt suggested most metabolic pathways were reduced in the skin, gills and intestine, particularly with a pronounced reduction in the intestine. As in the skin, the pathways “related to the cell division/cycle” and “bacterial secretion system” were significantly increased after *I. multifiliis* infection.

### 3.6. qRT-PCR Assays Indicated Expression of Mucosal Immune Marker Genes IgT and TNF-α Up-Regulated While Systemic IgM Fluctuated with Increasing Tendency After Infection

**Figure 7:**
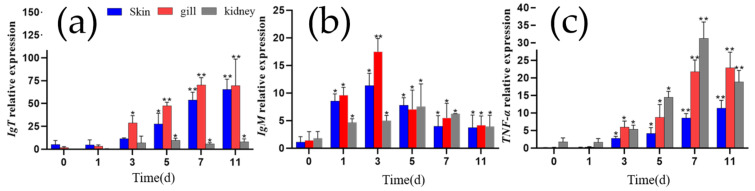
The relative expression levels of *IgT*, *IgM*, and *TNF-α* in the gills, skin, and kidney of grass carp. (**a**) *IgT*. (**b**) *IgM*.(**c**) *TNF-α*. Each column represents the relative expression levels of genes to β-actin, with the results expressed as the means ± standard error of three qRT-PCR assays (2^−ΔΔCT^). Differences between different infection days and day 0 were analyzed by one-way ANOVA, with * indicating significant differences (*p* < 0.05) and ** indicating highly significant differences (*p* < 0.01).

To investigate the changes in mucosal immune marker IgT, pro-inflammatory cytokine TNF-α, and systemic immune marker IgM expression in grass carp infected with *I. multifiliis*, the relative expression levels of the genes encoding IgT, TNF-α and IgM were analyzed in the gill, skin, and kidney tissues using qRT-PCR. Overall, the expression of the three genes was up-regulated in these three tissues following *I. multifiliis* infection. Specifically, *IgT* expression in the gill and skin increased significantly at all tested time points after infection (*p* < 0.05). *IgM* expression was significantly up-regulated in all three tissues at the early stages of infection (≤3 days), decreased after 3 days as the infection progressed but also maintained at higher levels than that before infection (*p* < 0.05). *TNF-α* expression in all three tissues also showed a significant increase with infection duration (*p* < 0.05) (Figure 7).

### 3.7. Microbiota Were Correlated with Biochemical and Immune Indicators

**Figure 8:**
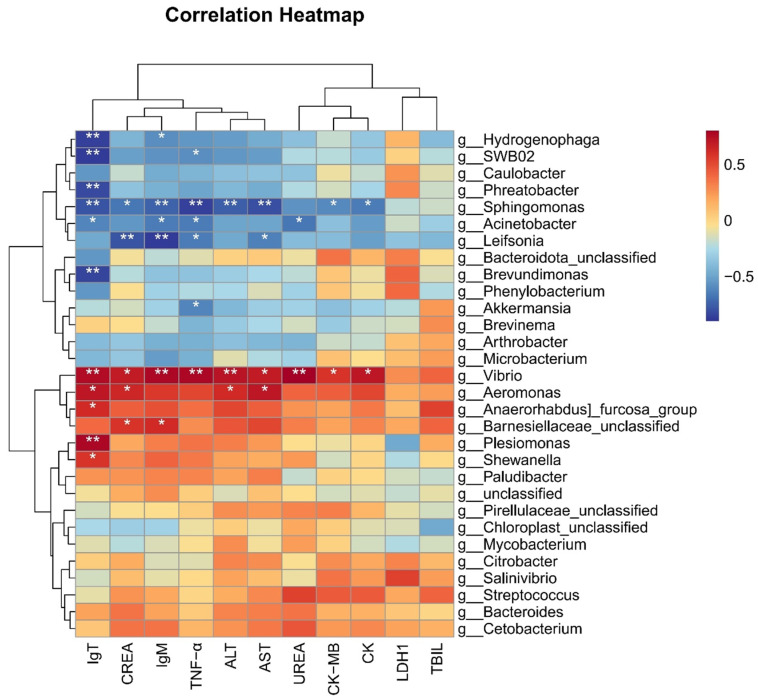
Correlation heatmap between microbes and phenotypes in grass carp. * indicates *p* < 0.05, ** indicates *p* < 0.01.

We selected the top 30 microbiota at the genus level based on their relative abundance and performed heatmap analysis with related immune and biochemical indicators. The heatmap analysis in this study revealed that the genus *Vibrio* exhibited significantly positive correlations with the six biochemical indicators, such as ALT, AST, CK, CK-MB, UREA, and CREA, and the three immune genes *IgT*, *TNF-α*, and *IgM* (Figure 8). Similarly, the genus *Aeromonas* showed a significant positive correlation with biochemical indicators such as ALT, AST, CREA, and immune gene *IgT* (Figure 8). These strong positive correlations suggest that an increase in the abundance of *Vibrio* or *Aeromonas* may be associated with impaired liver, kidney and cardiac/muscle functions, as well as an enhanced expression of immune genes *IgT*, *TNF-α*, and *IgM*.

Conversely, the heatmap analysis also indicated that the genus *Sphingomonas* was significantly negatively correlated with five biochemical indicators, such as ALT, AST, CK, CK-MB and CREA, and three immune genes *IgT*, *TNF-α*, and *IgM.* the genus *Acinetobacter* exhibited significant negative correlations with the three immune genes and UREA, while *Leifsonia* was significantly negatively correlated with *TNF-α*, *IgM*, CREA, and AST (*p* < 0.05). Additionally, the genera *Hydrogenophaga*, *SWB02*, *Phreatobacter*, *Brevundimonus*, and *Akkermansia* showed significant negative correlations with the expression of immune genes (Figure 8). These findings suggest that a reduction in the abundances of these microbial taxa may be linked to impaired liver, kidney, muscle/heart functions, as well as stimulated immune responses in grass carp.

## 4. Discussion

In this study, we systematically investigated the multifaceted physiological and microbial community changes in grass carp after infection with *I. multifiliis*. By analyzing biochemical indicators, histopathology, and skin/gill/gut microbiota, we discovered that infection caused significant liver and kidney damage in grass carp, as well as notable changes in the microbial communities in the skin, gills, and intestine. In addition, immune-related gene (*IgM*, *IgT*, and *TNF-α*) expression levels increased after infection with *I. multifiliis*. These findings not only further elucidate the complex effects of parasitic infection on host physiological health but also provide new perspectives for understanding the relationship among host–microbiota–parasite interactions. Compared to previous studies, this research is the first to apply 16S rRNA high-throughput sequencing technology to systemic investigate skin/gill/intestine microbiota in *I. multifiliis* infection grass carp. Our findings indicate that *I. multifiliis* infection not only directly affects the pathology of host tissues but may also exacerbate the proliferation of opportunistic pathogens by disrupting the ecological balance of microbial communities in the skin, gills, and intestine, thereby further affecting the host’s tissue damage and increasing immune response.

### 4.1. Pathological and Biochemical Changes

Ciliate parasites cause significant tissue damage in fish, particularly affecting skin and gill structures. In turbot (*Scophthalmus maximus*), infections correlate with high mortality rates characterized by skin swelling, gill necrosis, and inflammatory responses [30]. Rainbow trout (*Oncorhynchus mykiss*) infected with *I. multifiliis* exhibit epidermal thickening and increased mucus cell proliferation [4]. Severe *I. multifiliis* infection might lift the layers of integumental epithelium, accompanied by severe epidermal hyperplasia, cellular necrosis, and histolysis, in the species like goldfish (*Carassius auratus*), largemouth bass (*Micropterus salmoides*) and yellow catfish (*Pelteobagrus fulvidraco*) [17,18]. In this study, grass carp infected with *I. multifiliis* showed similar pathological changes, with parasite attachment and proliferation exacerbating host tissue damage, leading to epithelial cell hyperplasia and necrosis. Parasites may directly impair respiratory and oxygen exchange functions of fish gills and facilitate secondary opportunistic pathogen such as *Flavobacterium* invasion, which has been proved by this study of pathogen isolation and 16s rRNA sequencing. Additionally, severe infections were marked by the significant infiltration of inflammatory cells, particularly monocytes and lymphocytes, indicating a localized immune response. This was further supported by elevated expression levels of the mucosal immune gene *IgT* and the inflammatory cytokine *TNF-α* in this study.

Furthermore, in this study, we observed that *I. multifiliis* infection led to significant changes in several biochemical indicators of grass carp, which reflect the functions and health status of the liver and kidney. The activities of aspartate aminotransferase (AST), alanine aminotransferase (ALT), urea (UREA), and creatinine (CREA) were significantly elevated in the infected group. AST and ALT are typically released into the blood when liver cells are damaged, so elevated activities of AST and ALT are considered biomarkers of hepatic destruction [31,32,33]. Elevated levels of UREA and CREA in fish are often associated with damage to the renal tubules or glomeruli, which affect kidney function [34,35]. Studies have shown that infection of cod (*Gadus morhua*) with the nematode *Contracaecum osculatum* can cause severe liver damage [36] and fathead minnows (*Pimephales promelas*) infected with trematode *Ornithodipl ostomum* spp. exhibit significant increases in hepatic lipid peroxidation [37]. In this study, *I. multifiliis*, an ectoparasite found on gills and skin, caused hepatic and renal impairment despite its distant location from these organs. This internal damage may result from gill tissue disruption, affecting host respiration and energy metabolism. Additionally, microbiota changes, including opportunistic pathogens such as *Aeromonas*, likely induced secondary infections in internal organs, as confirmed by bacterial isolation and identification assays. Similarly to findings in common carp (*Cyprinus carpio*) infected with monogenean ectoparasites [38], grass carp infected with ciliate ectoparasite *I. multifiliis* showed elevated ALT, AST, CREA, and UREA levels in this study.

### 4.2. Gene Expression Levels of IgT, IgM, and TNF-α

In this study, we systematically evaluated the changes in the expression levels of immune-related genes *IgT*, *IgM*, and *TNF-α* in grass carp after infection with *I. multifiliis*. Relative quantitative PCR analyses revealed a significant up-regulation in the expression of both *IgT* and *TNF-α*, while the expression of *IgM* showed time-dependent fluctuations.

IgT, a fish-specific immunoglobulin, plays a well-established role in mucosal immunity. For example, in rainbow trout (*O. mykiss*) infected with *Ceratomyxa shasta* and *I. multifiliis*, the number of IgT^+^ B cells in mucosal tissues was significantly increased, and the concentration of IgT in mucus rose by several orders of magnitude [39]. At the transcriptional level, after *I. multifiliis* infection, *IgT* expression was notably higher in the skin and gills of rainbow trout (*O.mykiss*) and loach (*Misgurnus anguillicaudatus*) [40,41]. Similarly, the up-regulation of *IgT* transcription was observed in the skin of rohu (*Labeo rohita*) infected with *Argulus siamensis* and Nile tilapia (*Oreochromis niloticus*) infected with *Gyrodactylus cichlidarum* [42].

IgM plays a crucial role in activating systemic and mucosal adaptive immune responses against pathogenic challenges, including parasitic infections [43]. For instance, IgM expression was up-regulated in the spleen and skin of rainbow trout by the eighth day post infection with *I. multifiliis* [44]. Similarly, in channel catfish (*Ictalurus punctatus*) vaccinated with live *I. multifiliis* theronts, IgM levels in the gills and internal immune organs showed time-dependent fluctuations, aligning with our findings [45]. Comparable patterns were observed in Indian major carp (*Labeo rohita*), where IgM expression in the gills trended upward after infection with the ectoparasite *Dactylogyrus scorpius* [46].

Moreover, TNF-α, an important pro-inflammatory cytokine, reflects the host’s inflammatory response mechanisms triggered during infection. In helminth infections, TNF-α is involved in protective Th2 immune responses and promotes parasite expulsion. The expression levels of *TNF-α* were found to be up-regulated in goldfish (*Carassius auratus*) during *Gyrodactylus kobayashii* infection [47] and parasite-infected Tilapia nilotica (*O. niloticus*) [48]. These studies indicated that fish infected with ectoparasites may experience an increase in both innate and adaptive immune responses. PICRUSt-based functional predictions revealed that distinct alterations might be changed in microbial metabolic pathways that are associated with host immunity post infection. Further studies are needed to elucidate how the microbiota modulates host immune responses.

In conclusion, the significant up-regulation of *IgT* and *TNF-α* underscores the key roles of mucosal immunity and inflammatory responses in host defense, while the dynamic changes in *IgM* reflect the complexity of the systemic immune response.

### 4.3. Impact on Gill, Skin, and Intestinal Microbial Communities

In this study, we employed 16S rRNA high-throughput sequencing technology to systematically analyze the microbial communities in the skin, gills, and intestine of grass carp infected with *I. multifiliis*. The results demonstrated that infection significantly affected the diversity, structure, composition, and function of grass carp microbiota.

#### 4.3.1. Microbial Diversity

Our study revealed a significant decrease in the skin microbial diversity of grass carp following *I. multifiliis* infection. Similar patterns were observed in Atlantic salmon (*Salmo salar*), where parasitic infection reduced skin microbial diversity and destabilized community composition [49]. Likewise, parasitic infection in African elephantnose fish (*Gnathonemus petersii*) led to declines in biodiversity indices [50]. Gill and intestinal microbial communities showed non-significant decreases in diversity but showed decreasing trends; this may reflect that local microbial communities in gills and intestine were less affected than those in the skin.

#### 4.3.2. Phylum and Genus Abundance

In this study, after infection, the abundance of phylum *Proteobacteria* significantly increased in the gills, skin, and intestine. This phylum is often closely associated with inflammation. In ulcerative colitis and Crohn’s disease patients, increased *Proteobacteria* abundance correlated with heightened inflammation, suggesting the role in pouchitis and disease exacerbation [51,52,53]. Certain members of *Proteobacteria* are closely associated with inflammation and tissue damage, and their increased abundance may worsen parasitic infection effects. For example, intestine *Proteobacteria* expansion suggests infection promotes opportunistic pathogens like *Aeromonas*, intensifying pathological responses [54]. The increase in *Proteobacteria* in the intestine may indicate impaired intestinal barrier function post infection, making opportunistic pathogenic bacteria more prone to invasion and proliferation. This increase may further exacerbate the host’s pathological stress response and digestive dysfunction. Notably, a contrasting finding was reported by Zhang et al. [4], where they observed a decrease in the relative abundance of *Proteobacteria* in rainbow trout infected with *I. multifiliis*, differing from our observations. We hypothesize that these discrepancies could stem from variations in the immune systems, skin barriers between grass carp and rainbow trout, the inherently higher baseline proportion of *Proteobacteria* in the microbiota of grass carp, and a range of environmental factors such as water temperature, pH, and water quality management.

In this study, *Actinobacteriota* abundance was significantly decreased in the gills and intestine of the infected grass carp. *Actinobacteriota* typically includes beneficial microbes that help maintain the host’s immune balance [55]. The reduction in *Actinobacteriota* may weaken the protective role of the intestinal microbiota for the host. Some beneficial genus microbes belong to *Actinobacteriota* in the intestine and are closely related to host nutrition absorption and immune regulation, and their reduction may lead to intestinal microbial dysbiosis. Additionally, the decrease in *Deinococcota* in the infected group’s skin indicates their important protective role in parasitic infection. Some species within *Deinococcota* are resistant to high temperatures and radiation [56], so their reduction may indicate a weakened skin defense mechanism.

At the genus level, the microbial community changes were also substantial. In the gills, the abundance of the genus *Flavobacterium*, *Luteolibacter* and *Candidatus Megaira* significantly increased in the infected group (*p* < 0.01, *p* < 0.05 and *p* < 0.01, respectively). Notably, the genus *Flavobacterium* is widely studied for its pathogenicity and adaptability in aquatic environments. As an opportunistic pathogen, it causes fish disease symptoms such as gill damage, skin lesions, and necrotizing ulcers when the environment is suitable [57,58]. In this study, grass carp infected with *I. multifiliis* showed an increased abundance of *Flavobacterium* spp. in the skin as well as in the gills, indicating that parasitic infection promoted the invasion of this opportunistic pathogen, similarly with trout skin after parasitic infection [4]. *Luteolibacter* is a genus of Gram-positive bacteria, living in various environments including soil and freshwater [59]. However, there have been no reports to date on whether this bacterial genus is pathogenic or beneficial to fish. *Candidatus* Megaira, belonging to the order *Rickettsiales*, is a symbiotic bacterium in the environment, typically forming symbiotic relationships with ciliates, algae, and other prokaryotic or eukaryotic organisms. *Candidatus* Megaira has been found to possess potential for defensive symbiosis, with certain strains playing key roles in host protein interactions. *Candidatus* Megaira polyxenophila infection enhances host ciliate Paramecium growth performance, suggesting it plays a complex role in ecological adaptation [60]. In this study, the significant increase in *Candidatus* Megaira suggests that it might play a synergistic role in the ciliate parasitic infection process. Furthermore, whether the symbiotic genus *Candidatus* Megaira could serve as indicator species for ciliated parasitic infection also deserves a deeper exploration.

In the intestine, the genera *Aeromonas* and *Vibrio* were significantly increased in the infected group (*p* < 0.05), while *Brevundimonas* and *Hydrogenophaga* significantly decreased (*p* < 0.05). *Aeromonas*, well known as opportunistic pathogens, are frequently associated with diseases such as hemorrhagic septicemia, skin ulcers, and enteritis in aquatic animals, including grass carp, posing a significant threat to fish health [28]. In contrast, *Vibrio* primarily infects marine or brackish water fish [61]. In this study, *Aeromonas* were isolated as positive, while *Vibrio* were negative in grass carp. This observation might be attributed to the higher susceptibility of grass carp to *Aeromonas* than to *Vibrio* in freshwater environments, despite the presence of both bacterial taxa in the intestinal microbiota [61]. This study’s results indicate that *I. multifiliis* infection leads to an increase in opportunistic pathogens, consistent with previous research [4]. *Brevundimonas* is not a typical probiotic; its presence can enhance the stability of diverse microbial communities to improve the disease resistance of aquatic animals, thereby mitigating the impact of pathogenic bacteria. Additionally, *Brevundimonas* can promote the absorption and growth of teleosts by decomposing organic matter and releasing minerals and trace elements [62]. Although direct studies on the genus *Hydrogenophaga* in fish intestine are limited, existing characteristics suggest that they may promote intestinal health in various ways. Firstly, *Hydrogenophaga* has the ability to degrade organic compounds in the intestine, thereby aiding the host in better nutrient absorption [63]. Additionally, *Hydrogenophaga* can synergize with other beneficial microbial communities to maintain intestinal microbial balance and inhibit the proliferation of harmful pathogens, thereby reducing infection risks [64].

In the skin of the infected group, besides *Flavobacterium*, the abundance of the genus *Candidatus Midichloria* also significantly increased. *Candidatus Midichloria* is an intriguing group of endosymbiotic bacteria, widely distributed among various hosts, especially in ticks. The representative species *Candidatus Midichloria mitochondrii* is known for inhabiting host mitochondria and is considered the only bacterium capable of infecting mitochondria [65]. Additionally, other species in the *Midichloriaceae* family exhibit a broad host range, including aquatic invertebrates and protists, indicating the ecological diversity of this genus [66]. Further research is needed to understand why it significantly increased in the infected group.

### 4.4. Potential Microbial-Based Ciliate Parasitic Disease Management

This study demonstrated that the structure and function of the skin, gill, and intestinal microbiota in grass carp were significantly altered after infection with the ciliate ectoparasite *Ichthyophthirius multifiliis*. These changes differed from those induced by another ectoparasite, *Dactylogyrus lamellatus*, in grass carp [14]. While intestinal microbiota diversity similarly decreased after *Dactylogyrus* infection, the abundance of the opportunistic genus *Aeromonas* did not increase, though *Aeromonas* has been found to be in the top 10 bacterial genera in the intestine [14], which was different to the findings of this study. The observed differences may be attributed not only to the varying degrees of impact exerted by the parasites on the host but also to the host’s health status and immune-driven responses. Research has shown that fish immunity can sense and shape the intestinal microbiota [67]. In the study on *Dactylogyrus*-infected grass carp, serum pathological changes were less pronounced than those observed in this study with *I. multifiliis* infection, suggesting that *I. multifiliis* might pose a higher threat to grass carp health. The PICRUSt-based functional prediction of the microbiota revealed that after *I. multifiliis* infection, the metabolic function of the intestinal microbiota might be weakened, limiting its ability to provide adequate nutrition to the host. This may indirectly highlight the importance of enhanced nutritional management in aquaculture to improve resistance against parasitic infections.

Following *I. multifiliis* infection, the abundance of opportunistic pathogens such as *Aeromonas* and *Vibrio* in the intestine of grass carp increased significantly. This increase was positively correlated with elevated levels of liver and kidney functional markers (ALT, AST, and CREA), indicating exacerbated tissue damages. The findings suggest that controlling grass carp ichthyophthiriasis could mitigate secondary infections by pathogenic bacteria. Conversely, the reduction in beneficial bacteria such as *Brevundimonas* and *Hydrogenophaga* in the intestine may create an environment conducive to the over-proliferation of pathogenic bacteria. Further research is warranted on how to increase beneficial microbes in the intestine.

16S rRNA sequencing technology enables the detection of both culturable and unculturable bacteria, providing a more comprehensive profile of microbial communities compared to traditional isolation and cultivation methods. However, further in-depth research is needed to elucidate the functional roles of fish microbiota and their interactions with the host. In this study, Spearman’s rank correlation analysis was used to evaluate the relationships between phenotypic traits (biochemical and immune markers) and microbial communities in *I. multifiliis*-infected grass carp. This integrative approach enhances our understanding of the complex “host–microbiota–parasite” interactions. Future research should focus on exploring the functional roles of potential beneficial bacteria in preventing parasitic diseases. For instance, supplementing fish feed with these bacteria could be a promising strategy to prevent ichthyophthiriasis.

Interestingly, this study reveals an increase in the abundance of opportunistic pathogens, specifically *Aeromonas* and *Vibrio* in the gut and *Flavobacterium* in the gills and skin. These findings are significant for both researchers and farmers, suggesting that in future research on combating *I. multifiliis*, the use of oral probiotics to reduce the abundance of *Aeromonas* and *Vibrio* in the gut may be beneficial for controlling ichthyophthiriasis. Additionally, could cultured water disinfection and the application of probiotics targeting the abundance decrease in *Flavobacterium* also help to reduce *I. multifiliis* infections? These questions warrant further investigation. Moreover, whether the symbiotic genus *Candidatus* Megaira, which increased in the gills and skin, could serve as indicator species for ciliated parasites (such as *I. multifiliis*) also deserves a deeper exploration.

## 5. Conclusions

**Figure 9:**
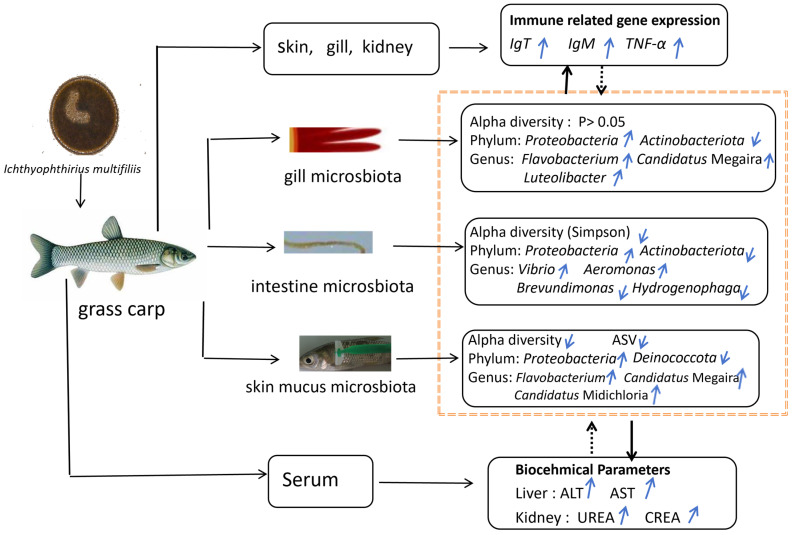
Graphical abstract of *I. multifiliis* infection in grass carp: effects on microbiota composition, biochemical parameters, and immune gene expression. Notes: black solid lines represent the mechanisms identified in this study. Black dashed lines represent deduced relationship. Blue upward arrows (↑) indicate increase, while blue downward arrows (↓) indicate decrease.

This study investigated the effects of *Ichthyophthirius multifiliis* infection on the microbial communities in the gills, skin, and intestine, serum biochemical indices, and immune responses in grass carp. The infection significantly altered the structure and composition of the host microbiota, reducing skin microbial diversity and increasing the abundance of opportunistic pathogens. Specifically, *Actinobacteriota* decreased in the gills and intestine, while *Proteobacteria* increased across the skin, gills, and intestine. The symbiotic genus *Candidatus* Megaira expanded in the gills and skin, while pathogenic genera *Flavobacterium* (gills/skin), *Vibrio* (intestine), and *Aeromonas* (intestine) proliferated markedly. These changes suggest that parasitic infection exacerbates host pathology by promoting opportunistic pathogen colonization and disrupting beneficial microbial symbiosis. Additionally, infected fish exhibited significantly elevated serum biochemical indices, including ALT, AST, UREA, and CREA. The up-regulation of mucosal immune markers (*IgT*) and pro-inflammatory cytokines (*TNF-α*) highlighted their critical role in host defense mechanisms. Meanwhile, *IgM* expression showed time-dependent fluctuations with an overall increasing trend, reflecting the complexity of the immune response. Microbial alterations were closely correlated with serum biochemical parameters and immune-related gene expression, particularly *Vibrio* and *Aeromonas*, which showed strong positive correlations with ALT, AST, and CREA levels. These findings provide insights into “host–microbiota–parasite” interactions and offer potential microbial-based strategies for controlling parasitic diseases in aquaculture (Figure 9).

## Figures and Tables

**Table 1:** Primers used to determine relative expression of immune-related genes.

Primer Name	Sequence (5′–3′)	GenBank Accession No.
*β-actin*	F: 5′-GGATGATGAAATTGCCGCACTGG-3′	M25013
	R: 5′-ACCGACCATGACGCCCTGATGT-3′	
*IgT*	F:5′-AGGAGGAGGTCTGGAGTG-3′	GQ201419.1
	R:5′-GAAAGCCCAGTTGTAGTT-3′	
*TNF-α*	F: 5′-TGTGCCGCCGCTGTCTGCTTCACGCT-3′	NC067238
	R: 5′-GATGAGGAAAGACACCTGGCTGTAGA-3′	
*IgM*	F:5′-TCTACCTCCAACTCCACCACC-3′	DQ417927
	R:5′-TGTTTATTGTATTTGCCACCTGAT-3′	

**Table 2:** Pathogen detection results in uninfected and infected grass carp.

Pathogen	Uninfected Group	Infected Group
*I. multifiliis*	−	+++
GCRV	−	−
*Flavobacterium*	−	+
*Aeromonas*	−	+
*Vibrio*	−	−

“−” indicates negative, “+ ~ +++ ” indicates positive and the infected degree from mild to severe.

**Table 3:** Biochemical indicators of grass carp (*Ctenopharyngodon idellus*) infected and uninfected with *I. multifiliis*.

Parameters	Uninfected Group	Infected Group
ALT (U/L)	4.25 ± 2.40	12.20 ± 6.89 *
AST (U/L)	60.95 ± 18.50	234.65 ± 151.22 *
TBIL (μmol/L)	7.63 ± 1.30	11.63 ± 6.60
UREA (mmol/L)	0.54 ± 0.24	1.01 ± 0.37 *
CREA (μmol/L)	19.34 ± 3.86	35.06 ± 14.52 *
CK (U/L)	3148.58 ± 1411.64	6661.09 ± 4007.94
CK-MB (U/L)	2562.24 ± 1118.26	4988.95 ± 2963.95
LDH1 (U/L)	141.22 ± 56.47	165.72 ± 88.19

Note: Values are presented as means ± SD. * Means in the same line denote a significant difference (*p* < 0.05). ALT: alanine aminotransferase; AST: aspartate aminotransferase; TBIL: total bilirubin; UREA: urea; CREA: creatinine; CK: creatine kinase; CK-MB: creatine kinase-MB; LDH1: lactate dehydrogenase 1.

**Table 4:** Alpha diversity of skin, gill, and intestine microbiota community in grass carp infected with *I. multifiliis* and in control group.

Groups	Tissue	Observed Species	Shannon	Simpson	Chao1	Goods Coverage
Control	Skin	452.00 ± 10.12	5.38 ± 0.33	0.86 ± 0.05	455.19 ± 15.45	1
	Intestine	177.33 ± 12.45	2.88 ± 0.45	0.65 ± 0.04	177.68 ± 13.65	1
	Gill	341.00 ± 10.12	3.93 ± 0.33	0.70 ± 0.05	341.02 ± 15.45	1
Infection	Skin	280.17 ± 12.45 *	3.64 ± 0.45 *	0.73 ± 0.04	281.92 ± 13.65 *	1
	Intestine	172.50 ± 10.12	2.57 ± 0.33	0.52 ± 0.05 *	173.04 ± 15.45	1
	Gill	321.50 ± 12.45	3.37 ± 0.45	0.68 ± 0.04	321.62 ± 13.65	1

Note: * indicates a significant difference compared to the control group (*p* < 0.05).

## Data Availability

All 16S rRNA genes of microbiota sequences were deposited in the NCBI Sequence Read Archive under BioProject PRJNA1224981. http://www.ncbi.nlm.nih.gov/bioproject/1224981 (accessed on 18 February 2025).
